# Effects of *AMPD1* common mutation on the metabolic-chronotropic relationship: Insights from patients with myoadenylate deaminase deficiency

**DOI:** 10.1371/journal.pone.0187266

**Published:** 2017-11-02

**Authors:** Fabrice Rannou, Virginie Scotet, Pascale Marcorelles, Roxane Monnoyer, Cédric Le Maréchal

**Affiliations:** 1 Physiology Department-EA 4324, CHRU Cavale Blanche, Brest, France; 2 Institut National de la Santé et de la Recherche Médicale - UMR 1078, Brest, France; 3 Pathology Department-EA 4685 LNB, CHRU Morvan, Brest, France; Freeman Hospital, UNITED KINGDOM

## Abstract

**Purpose:**

Current evidence indicates that the common *AMPD1* gene variant is associated with improved survival in patients with advanced heart failure. Whilst adenosine has been recognized to mediate the cardioprotective effect of C34T *AMPD1*, the precise pathophysiologic mechanism involved remains undefined to date. To address this issue, we used cardio-pulmonary exercise testing data (CPX) from subjects with myoadenylate deaminase (MAD) defects.

**Methods:**

From 2009 to 2013, all the patients referred in our laboratory to perform a metabolic exercise testing, i.e. a CPX with measurements of muscle metabolites in plasma during and after exercise testing, were prospectively enrolled. Subjects that also underwent an open muscle biopsy for diagnosis purpose were finally included. The metabolic-chronotropic response was assessed by calculating the slope of the linear relationship between the percent heart rate reserve and the percent metabolic reserve throughout exercise. MAD activity was measured using the Fishbein’s technique in muscle biopsy sample. The common *AMPD1* mutation was genotyped and the *AMPD1* gene was sequenced to screen rare variants from blood DNA.

**Results:**

Sixty-seven patients were included in the study; 5 had complete MAD deficiency, 11 had partial MAD deficiency, and 51 had normal MAD activity. Compared with normal MAD activity subjects, MAD deficient subjects appeared to have a lower-than-expected metabolic-chronotopic response during exercise. The metabolic-chronotropic relationship is more closely correlated with MAD activity in skeletal muscle (Rs = 0.57, *p* = 5.93E-7, Spearman correlation) than the presence of the common *AMPD1* gene variant (Rs = 0.34, *p* = 0.005). Age-predicted O_2_ pulse ratio is significantly increased in MAD deficient subjects, indicating a greater efficiency of the cardiovascular system to deliver O_2_ (*p* < 0.01, Scheffé’s post hoc test).

**Conclusion:**

The metabolic-chronotropic response is decreased in skeletal muscle MAD deficiency, suggesting a biological mechanism by which *AMPD1* gene exerts cardiac effect.

## Introduction

The clinical course and long-term outcome of patients with chronic heart failure (CHF) vary widely among individuals. In recent years, the genotype background has received increased attention owing to the existence of modifier genes that can modulate the severity and progression of heart diseases [1]. In 1999, using Kaplan-Meier survival-time plot analysis, Loh *et al*. first reported in end-stage cardiomyopathy a significantly greater probability of survival associated with carriage of the *AMPD1* gene common mutation [2]. *AMPD1* codes for the skeletal muscle isoform of myoadenylate deaminase (MAD). MAD promotes the deamination of adenosine monophosphate (AMP) to inosine monophosphate (IMP). A common mutation in the *AMPD1* gene second exon (so-called C34T) in the homozygous state led to a truncated, catalytically inactive enzyme [3]. As a result, MAD deficiency is the most common enzymatic defect of skeletal muscle [3–7]. Despite intense study, the exact pathophysiological mechanism behind the beneficial effect of *AMPD1* common mutation on cardiac function remains elusive. Nonetheless, there is ample evidence indicating that adenosine contributes significantly to the cardiovascular effects of C34T *AMPD1* [8–10].

Due to MAD defect, AMP accumulates in muscle and the alternative pathway for AMP catabolism is to form adenosine by dephosphorylation via two different 5'-nucleotidases, the ecto-5'-nucleotidase and the AMP-selective cytosolic 5'-nucleotidase [11]. During exercise, the production of adenosine is therefore increased several fold in MAD deficient patient [8, 9], leading to subsequent positive effects on cardiovascular function. Adenosine has been shown to promote a beneficial vasodilatation for coronary blood supply [12], anti arythmic effect [10, 12, 13]. Further, it has also been shown that adenosine exerts negative chronotropic response via sinus atrial [12], even in the presence of catecholamines as during exercise [14]. An unresolved issue is that it is not clear whether the beneficial effect of adenosine on cardio-vascular function is linked to C34T or the muscle AMP activity. Patients with known MAD deficiency may provide a useful knock-out model of *AMPD1* defect to study the effects of adenosine on cardio-vascular function during exercise [4].

During incremental exercise, the heart rate increase (i.e., chronotropic response) is strongly correlated with oxygen consumption increase. To calculate this linear relationship between the chronotropic and metabolic responses during dynamic incremental exercise (metabolic-chronotropic relationship, MCR), Wilkoff *et al*. have proposed a mathematical model [15]. The strength of this model is that it allows comparison between subjects irrespective of gender and anthropometric data, and is reliable even in case of submaximal exercise.

Consequently, the purpose of this study was twofold. First, we hypothesized that the increase in adenosine production in MAD deficient patients modulates their chronotropic response during exercise. Second, we sought to examine the respective effects of *AMPD1* common mutation and MAD deficiency on MCR by using cardio-pulmonary exercise testing (CPX) data from subjects with known MAD activity (i.e., Absent, Decreased, and Normal) in skeletal muscle.

## Materials and methods

### Design and subjects

This was an observational study performed at the Physiology Department of Brest Hospital from May 2009 to December 2013 in patients with exercise-related myalgia. All the patients (age > 16 years) referred in our laboratory to perform a metabolic exercise testing, i.e. a CPX with measurements of muscle metabolites in plasma during and after exercise testing (for more details, see [16]), were prospectively enrolled. In this cohort, subjects that also underwent an open muscle biopsy for diagnosis purpose were finally included. Exclusion criteria were as follows: (a) history of heart disease, neuropathy (b) use of ß-blocking agents, digitalis, calcium channel blockers, amiodarone, (c) arrhythmias preventing proper HR assessment. Furthermore, the presence of diabetes mellitus and thyroid dysfunction were ruled out by medical history, physical examination, and measurement of fasting glycemia and TSH.

A group of eighteen healthy age-matched individuals, referred to undergo CPX to assess their physical fitness in our department, were recruited as controls for comparison of exercise responses. Controls were sedentary or moderately active subjects, with no history of muscle or cardiovascular diseases, and their physical examination was normal. Other exclusion criteria were similar to those used for the patients with exercise-induced myalgia.

The study conforms to the principles outlined in the Declaration of Helsinki. The Institutional Ethics Committee at Brest University Medical Center approved the study. Each participant, and their legal representatives if they were minor, completed a written informed consent prior to participation (Clinical Trial NCT02362685).

### Exercise protocol

All tests were performed in the morning after an overnight fast. Subjects were requested to refrain from exercise in the 48 hours before the study and from caffeine consumption on the day of the test. Each subjects performed a symptom-limited cardio-pulmonary exercise testing (CPX) on and electronically braked cycle ergometer (Ergoline GmbH, Bitz, Germany). Gas exchange was measured breath-by-breath using a MedGraphics CPX gas exchange system (Medical Graphics Corporation, St. Paul, Minnesota). The pneumotachometer and the O_2_ and CO_2_ analyzers were calibrated and the environmental temperature, percent humidity, and barometric pressure updated before each exercise session. The surface ECG was continuously monitored an blood pressure was measured every 2 min. Resting oxygen consumption and heart rate were calculated as the mean value during the last 2 minutes prior to starting exercise. In order to exhaust each subject’s limit of tolerance within 8–12 min, the predicted maximal power (PMP) was determined according to anthropometric data [17–18]. The test started with an initial 2-min workload of 20% PMP, with an increment of 10% PMP per minute. All patients were verbally encouraged to reach volitional exhaustion.

The predicted peak VO_2_ values were determined according to the equations proposed by Wasserman and Hansen [19]. Percent-predicted maximal power was calculated according to normative values proposed by Jones [18].

The MCR, also referred to as chronotropic index, was calculated from the ratio of the heart rate reserve (HRR) to the metabolic reserve (i.e. V’O_2_ reserve) throughout incremental exercise [15]. The percent heart rate reserve (%HRR) at each stage of exercise was calculated as follows [15]:

$$\%HRR=\frac{HR_{stage}-HR_{rest}}{HR_{max}-HR_{rest}}$$

Where HR_stage_ is the heart rate at a given stage; HR_rest_ is the heart rate at rest, calculated as the average of heart rate during the 2-min rest period before exercise; HR_max_ is the age predicted maximal heart rate given by 220-age (in years).

The percent metabolic reserve (%V’O_2_ reserve) was calculated as follows [15]:

$$\%V\prime O_{2reserve}=\frac{V\prime O_{2stage}-V\prime O_{2}{}_{rest}}{V\prime O_{2}{}_{max}-V\prime O_{2}{}_{rest}}$$

Where V’O_2stage_ is the oxygen consumption at a particular stage; V’O_2 rest_ and V’O_2 max_ are the resting and the maximal oxygen consumptions, respectively.

Data were plotted as %HRR versus %V’O_2 reserve_ at each 30s exercise from rest to peak exercise. The slope of the linear relationship (reserve slope) was computed for each subject by linear regression analysis.

Peak O_2_ pulse was expressed as a percentage of the predicted value achieved by dividing predicted peak V’O_2_ by the predicted maximal heart rate [20]. Forehead pulse oximetry (Nonin 8000R, Nonin Medical, Inc., Plymouth, MA) was used to monitor arterial O_2_ saturation [21]. Hemoglobin concentration was determined before exercise from venous blood sample.

### *AMPD1* gene testing

Genomic DNA was extracted from whole blood. The genotyping was restricted to the main single nucleotide mutation of the *AMPD1* gene (rs17602729) previously named C34T in most previously published studies. The nucleotide numbering starts to the A of the first translated codon according to the Human genome variation society nomenclature based on the reference transcript NM_000036.1. Genotyping was performed by 5’-nuclease assay with MGB probes (TaqMan SNP genotyping assay thermo Fisher, ref. C__33603912_10) on a Lightcycler 480 (Roche) system. Sanger sequencing was performed to look for rare variants within the 16 exons and their flanking introns (reference sequence NM_000036.2). Bigdye terminator v1.1 cycle sequencing kit (ThermoFisher) was used and the sequences were aligned with SeqPilot software (JSI Medisys; sequencing primers available upon request).

### MyoAdenylate deaminase activity measurement

Histo-enzymology was performed on fresh-frozen muscle biopsies from deltoid or vastus lateralis. MAD activity was demonstrated by the incubation (22 ± 2°C for 1 hour) of muscle transverse sections in Fishbein’s medium containing substrate (adenosine monophosphate, 1.2 M) and an electron acceptor (p-nitro-blue tetrazolium, 3.2 mM), potassium chloride (0.2 M), and dithiothreitol (0.1 M) at pH 6.1. After staining, the sections were rinsed with distilled water and mounted in glycerin jelly.

The end-product of the Fishbein’s technique (i.e. *p*-NBT formazan) gives a blue staining with a wide absorption spectrum. MAD activity determinations were made using digitized stained sections as blue-level images (Olympus BX51 color camera, Hamburg, Germany). The optical density (OD) of MAD activity-staining was calculated for each section (Mesurim Pro Software, jean-francois.madre@ac-amiens.fr) according to the equation:

$$OD=100*{\text{log}}_{10}(\frac{I_{o}}{I_{x}})$$

Where I_o_ is the measured intensity of light transmitted through slide, cover-slip and mounting medium in areas immediately adjacent to the muscle section, and I_x_ is the intensity of light transmitted through muscle tissue. To provide an objective judgment of relative staining intensity, OD was compared to stains from the same series, namely biopsy samples from other patients processed at the same time [16]. For the purpose of comparing the MCR according to *AMPD1* C34T genotype and MAD activity, the latter was divided in three subgroups (Normal, Decreased, Absent) as previously reported [6,16,22].

### Statistics

The data were first analyzed for normality and homogeneity of variances by using Shapiro-Wilk and Bartlett tests (95% confidence interval), respectively. Intergroup comparison was carried out using one-way ANOVA, followed by Scheffé’s post hoc analysis for multiple comparisons. The genotype-phenotype relationship between *AMPD1* C34T and MAD activity can be formulated in a 3 x 3 contingency table. Agreement between *AMPD1* C34T and MAD activity was evaluated using the weighted kappa statistics. Associations between MCR and *AMPD1* C34T genotype, and between MCR and MAD activity, were measured using Spearman’ rank correlation coefficient (Rs). To assess whether any differences between these non independent Spearman’rank correlation magnitudes were statistically significant, we applied the Williams-Steiger’ t-test [23]. Data are reported as mean and standard deviation of the mean (SD). A *p* value of less than 0.05 (95% confidence level) was considered significant (SAS software, ver. 9.2).

## Results

We prospectively enrolled 74 patients, of which 7 subjects were excluded from statistical analysis because of an alternative diagnosis at muscle biopsy (glycogenosis and respiratory chain deficiency, n = 5 and 2, respectively). Complete MAD deaminase deficiency was found in 5 patients, partial MAD deaminase deficiency enzyme activity in 11 subjects, and normal MAD activity in 51 subjects.

The distribution of base-line characteristics and CPX data in subjects is presented in Table 1. Partial or complete MAD deficiency has little impact on maximal O_2_ consumption (% predicted peak V’O_2_; *p* = 0.30, ANOVA). Conversely, the maximal heart rate at peak exercise was significantly decreased in the two MAD defect subgroups (% Predicted Maximal Heart Rate; *p* = 2.76 E-08, ANOVA). Although significant differences in RER at exhaustion were detected among groups by ANOVA, the inter-group post-hoc analysis did not reveal significant differences.

**Table 1:** Demographics data and exercise test responses according to skeletal muscle MAD activity and in controls subjects.

	Absent	MAD activity Decreased	Normal	Control	ANOVA F-value (*p*-value)
**Number (n)**	5	11	51	17	
**Sex (f/m)**	2/3	9/2	9/42	9/8	
**Age (years)**	32.6 ± 18.7	40.2 ± 13.3	35.8 ± 13.0	36.5 ± 11.3	0.49 (0.69)
**BMI (kg.m**^**-2**^**)**	27.5 ± 10.0	22.6 ± 4.5	23.9 ± 4.3	25.4 ± 5.3	1.50 (0.22)
**Hemoglobin (g.dL**^**-1**^**)**	14.5 ± 1.1	13.8 ± 0.9	14.6 ± 1.1	14.2 ± 1.0	1.88 (0.14)
**Resting Systolic BP (mmHg)**	124.2 ± 19.5	111.3 ± 15.7	114.4 ± 16.1	115.6 ± 14.3	0.79 (0.51)
**Resting Diastolic BP (mmHg)**	76.2 ± 17.9	68.9 ± 12.1	73.7 ± 11.2	72.8 ± 12.0	0.61 (0.61)
**Peak exercise Systolic BP (mmHg)**	176.4 ± 19.9	172.1 ± 29.4	170.0 ± 26.8	166.2 ± 20.8	0.25 (0.83)
**Peak exercise Diastolic BP (mmHg)**	83.6 ± 17.2	79.2 ± 17.0	83.0 ± 11.7	82.8 ± 14.2	0.26 (0.85)
**% Predicted maximal power**	75.4 ± 23.0	90.9 ± 22.0	95.1 ± 21.6	98.8 ± 18.0	1.72 (0.17)
**Peak V’O**_**2**_ **(ml.min**^**-1**^.**kg**^**-1**^**)**	30.9 ± 16.3	30.9 ± 11.6	36.5 ± 10.4	32.1 ± 6.7	1.61 (0.19)
**% Predicted peak V’O**_**2**_	87.8 ± 23.4	100.6 ± 17.4	98.4 ± 15.8	103.3 ± 14.7	1.25 (0.30)
**Heart Rate at end-exercise (beats/min**^**-1**^**)**	137.6 ± 22.0	150.3 ± 23.7	172.3 ± 16.8 *†	175.4 ± 11.0 *†	11.21 (3.22E-06)
**% Predicted Maximal Heart Rate**	73.2 ± 6.2	83.4 ± 10.6	93.6 ± 7.6 *†	95.6 ± 6.2 *†	16.12 (2.76E-08)
**RER at peak exercise**	1.10 ± 0.12	1.17 ± 0.09	1.21 ± 0.08	1.22 ± 0.07	3.17 (0.03)
**Pulse oximetry at peak exercise (%)**	97.8 ± 2.3	98.6 ± 1.0	97.6 ± 1.4	97.9 ± 1.0	1.56 (0.21)
**Metabolic-Chronotropic Relationship**	0.54 ± 0.12 ‡	0.75 ± 0.13	0.91 ± 0.13*†	0.93 ± 0.12*†	18.04 (4.9E-09)
**% Predicted maximal O**_**2**_ **pulse**	119.0 ± 26.7	120.1 ± 9.1	105.1 ± 16.2 ‡	107.9 ± 13.5	3.60 (0.02)

MyoAdenylate Deaminase, f female, m male, BMI Body Mass Index, BP Blood Pressure, RER Respiratory Exchange Ratio. Data are means ± SD. Comparisons between groups were made using analysis of variance and Scheffe’s post-hoc test. Data are presented as mean ± SD.

* *p* < 0.001 versus MAD Absent.

**^†^** *p* < 0.01 versus MAD Decreased.

^‡^ *p* < 0.05 versus MAD Decreased.

Table 2 presents the genotype-phenotype relationship between *AMPD1* c34T and MAD activity. Four patients carried the *AMPD1* C34T mutation in homozygosity, and 12 patients were heterozygotes. The weighted kappa coefficient between *AMPD1* C34T and MAD activity was 0.76 with 95% CI (0.60, 0.93).

**Table 2:** Confusion matrix of MAD activity in skeletal muscle vs. the most common *AMPD1* mutation in patients.

	Normal MAD activity	Decreased MAD activity	Absent MAD activity
**C34T Wild-Type**	46	5	
**C34T heterozygous**	5	6	1
**C34T homozygous**			4

Sequence analysis of the entire coding region of *AMPD1* revealed less frequent mutations in the present cohort (Table 3). Analysis of exon 7 revealed the presence of the A860T mutation in four subjects (patients 1–4). One subject was A860T homozygous (patient 2), two subjects were simple heterozygote (patients 1 and 3), and one subject was C34T/A860T compound heterozygote (patient 4). A 56-yr-old subject with absent MAD activity in skeletal muscle (patient 5) was found to be compound heterozygous for the C34T and G468T mutations. Unfortunately, parental DNA was not available to determine allele segregation and location of the two mutations.

**Table 3:** *AMPD1* rare variants carriers phenotype for the metabolic-chronotropic relationship and the predicted maximal O_2_ pulse.

Patient No.	MAD activity	*AMPD1*		Metabolic-Chronotropic Relationship	% Predicted maximal O_2_ pulse
		C34T Genotype	Rare variants (ref seq NM_000036.1)		
**1**	Normal	Wild-Type C/C	c.860A>T-p.K287I Heterozygote	0.927	95.8
**2**	Normal	Wild-Type C/C	c.860A>T-p.K287I Homozygote	0.796	112.0
**3**	Decreased	Wild-Type C/C	c.860A>T-p.K287I Heterozygote	0.730	126.4
**4**	Decreased	Heterozygote C/T	c.860A>T-p.K287I Heterozygote	0.744	114.7
**5**	Absent	Heterozygote C/T	c.468G>T-p.Q156H Heterozygote	0.511	155.2

**Fig 1:**
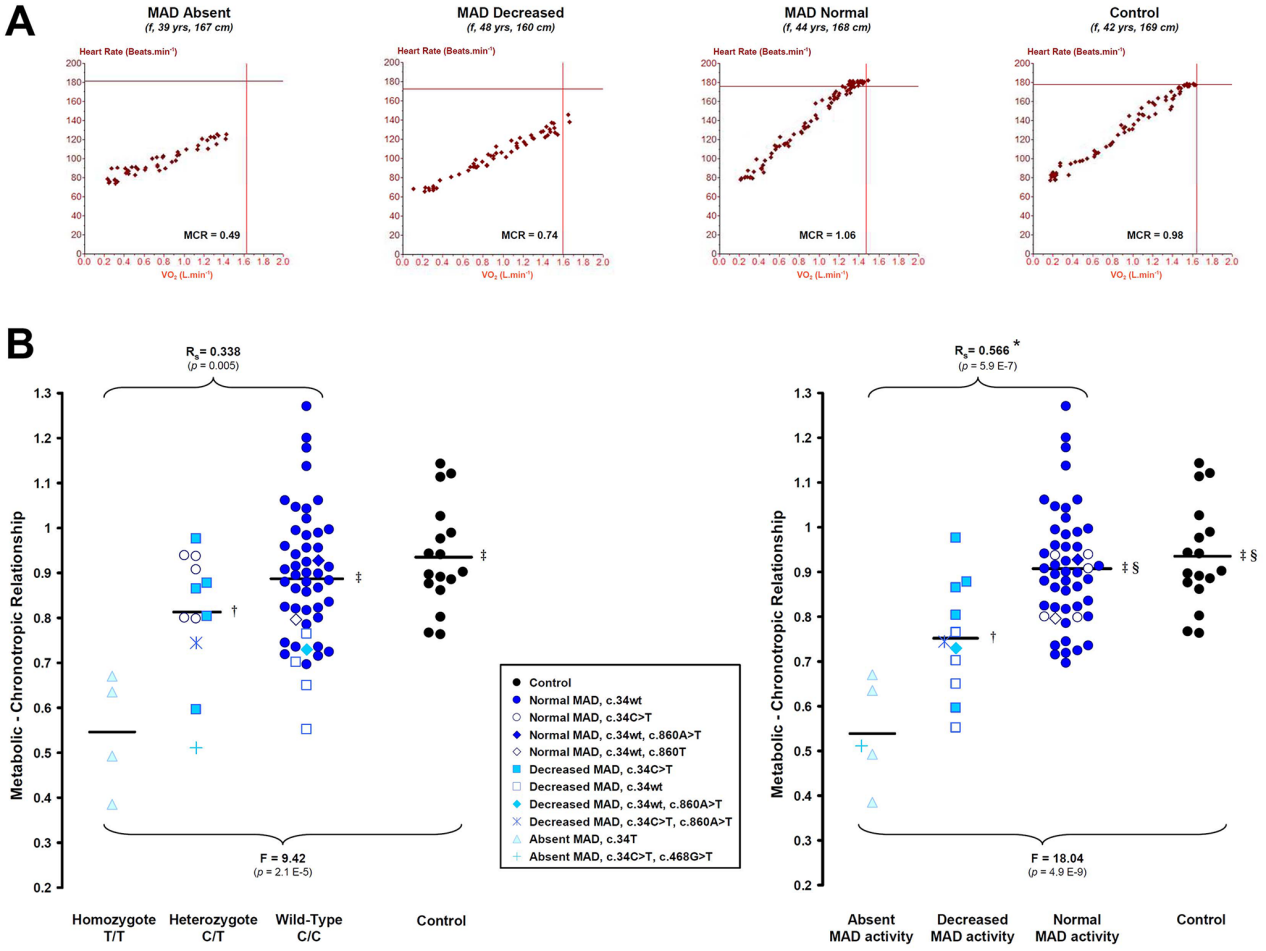
Effects of *AMPD1* C34T and MAD activity on the metabolic-chronotropic relationship. A Representative plots of the heart rate versus oxygen consumption according to MAD activity in three patients and in one control subject. For comparison, the four subjects have similar anthropometric data, i.e. similar predicted values for maximal exercise testing data. f, female. MCR, metabolic-chronotropic relationship. B Scatter plots reporting the metabolic-chronotropic relationship according to the *AMPD1* C34T genotype (left panel) and the MAD activity (right panel). Horizontal bars represent the mean. Rs, Spearman’s rank correlation coefficient along with associated two-tailed *p* value. *Significantly different from the C34T-genotype spearman correlation coefficient (Williams-Steiger’ t-test, *p* = 0.015). Intergroup comparison was performed by one way ANOVA followed by the Scheffe post hoc test. **†** significantly different from MAD Absent (*p* < 0.05). ‡ significantly different from MAD Absent (*p* < 0.001). § significantly different from MAD Decreased (*p* < 0.01).

Fig 1 displays the effects of *AMPD1* C34T and MAD activity on the MCR. As previously, we first sought to characterize our phenotype according to the previously and widely used methodology (i.e., *AMPD1* C34T genotype, Fig 1B left panel). The spearman correlation coefficient (R_s_) between the MCR and the *AMPD1* C34T genotype was 0.34 (*p* = 0.005), and between the MCR and MAD activity was 0.57 (*p* = 5.93E-7). Williams' t-test revealed a significant statistical difference between these two dependent correlation (*p* = 0.015).

**Fig 2:**
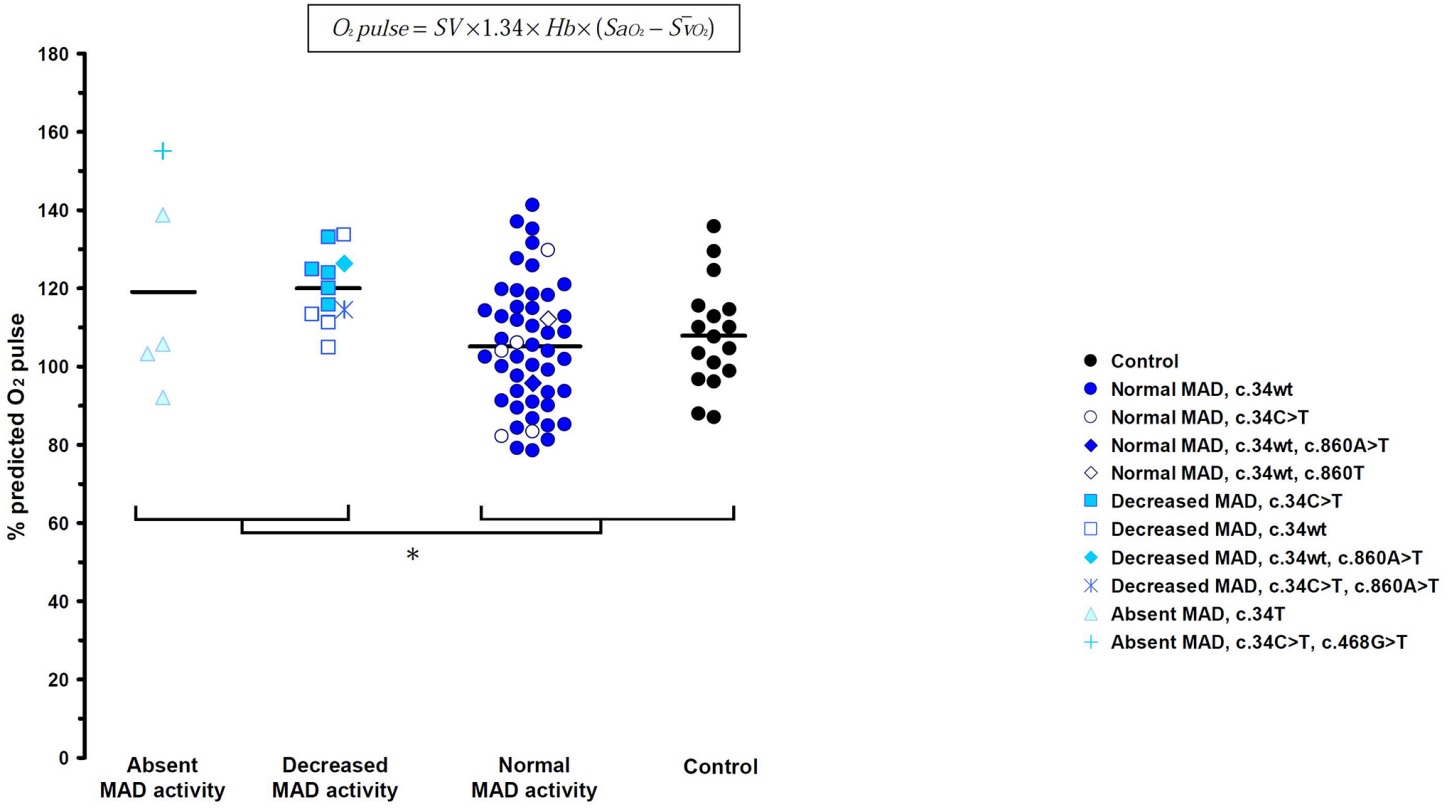
Effects of MAD activity on the % age-predicted O_2_ pulse. Horizontal bars represent the mean.* MAD defect (Absent MAD activity + Decreased MAD activity) significantly different from normal (Normal MAD activity + Control) according to the Scheffé grouping post hoc test (*p* < 0.01). Inset, O_2_ pulse is determined by stroke volume (SV), hemoglobin content (Hb) and arteriovenous oxygen saturation difference.

Such difference in the metabolic-chonotropic response according to MAD activity prompted us to analyse the predicted oxygen pulse ratio (Fig 2). ANOVA revealed significant differences among the groups (F = 3.60, *p* = 0.017). The post hoc contrasts according to the Scheffé method rejected the null hypothesis positing (mean _*MAD Absent*_ + mean _*MAD Decreased*_) − (mean _*MAD Normal*_ + mean _*Control*_) = 0 (*p* < 0.01), indicating that % age-predicted O_2_ pulse ratio is significantly increased in MAD defect (Absent and Decreased MAD activity).

## Discussion

The seminal paper by Loh and colleagues [2] attracted tens of subsequent studies to identify the mechanisms underlying the beneficial effects of *AMPD1* common variant on cardiovascular function. To address this issue, a significant amount of work has been devoted to studying the effects of C34T on clinical outcome of patients with CHF. In order to elucidate further this missing link in the genotype-phenotype relationship, we used CPX data from subjects whose MAD activity in skeletal muscle and C34T *AMPD1* status were evaluated concurrently. Furthermore, it should be emphasized that cardiac patients are given rhythm modulator drugs, thereby altering their chronotropic response during exercise. Hence, the interest to study subjects free from cardiovascular disease and with known MAD activity in skeletal muscle.

In the present study, we found 1) MAD deficient subjects have a lower metabolic-chronotropic response during exercise, and 2) this decrease response is more correlated to MAD activity in skeletal muscle compared to the C34T *AMPD1* genotype.

The beneficial effect of C34T on cardiovascular function was not observed in all studies [2,24–26]. Bearing in mind the non-linearity of the genotype-phenotype relation for *AMPD1* C34T and MAD activity, we hypothesize that the equivocal findings reported in the literature may relate to the methodology in previous studies. The segregation of patients into two classes (*AMPD1* C34T carriers vs. non carriers), rather than in three classes, may result in loss of statistical information. Less common *AMPD1* G468T and A860T mutations can also alter MAD activity. The A860T mutation produces an amino acid substitution of lysine for leucine at residue 2871 of MAD enzyme, resulting in an approximately 50% loss of catalytic activity [27]. The G468T mutation results in a substitution of glutamine for histidine [28]. In this study, the patient harboring the combined mutation G468T/C34T in the heterozygous state exhibited an absence of MAD activity in muscle biopsy, in agreement with previous reports [28, 29].

As originally described by Wilkoff *et al*. [15], we used the 220-age equation to predict maximal heart rate and calculate the MCR. There is an ongoing debate about the use of Tanaka equation (208–0.7*age) rather than 220-age formula to predict maximal heart rate [30, 31]. In our data set, the two equations yield strikingly similar results, with a difference less than 0.5% in the different groups (data not shown). The consistency of the results probably lies in the age of our studied population, since the two equations produce the same results between 30 and 40 years old (see Fig 3 in [30]). It has long been recognized that during increasing exercise workloads, heart rate is linearly related to the rate of oxygen consumption. This strong physiological property was used by Wilkoff and Miller to develop their mathematical model [15]. Wilkoff and co-workers originally designed their model of the MCR in order to, first, objectively measure a chronotropic incompetence in heart disease patients and, second, to assess the beneficial effect of pace-maker implantation on heart-rate response during exercise. According to the Wilkoff’s model, the MCR slope of the normal sinus is 1.0 with a 95% confidence interval between 0.8 and 1.3 [15]. In heart failure patients, an MCR slope of < 0.8 is considered indicative of chronotropic incompetence. Since subjects were free from cardiovascular disorders in this study, the lower metabolic chronotropic response in MAD deficient subjects should not be interpreted as chronotropic incompetence. Thus, our findings raise substantial and attractive issues from a physiological–“Fickian”- perspective.

While MCR provides an assessment of the relationship between oxygen consumption and heart rate during exercise, the ratio of the maximal values of these two variables (O_2_ pulse) also provides valuable insights. According to the Fick equation, O_2_ pulse is numerically equal to the product of stroke volume (SV) and arteriovenous O_2_ content difference (CaO_2_- CvO_2_). During incremental exercise, the increase in oxygen pulse is due to both an increase in stroke volume and a widening of the arterial-venous oxygen concentration difference (Fig 2 inset). Since the hemoglobin content was similar among the groups and no desaturation was observed in the study population using pulse oximetry, the arterial content in O_2_ does not account for the higher O_2_ pulse values in MAD deficient patients. The higher O_2_ pulse values in MAD deficient subjects are therefore due to either or both a greater stroke volume or a greater oxygen extraction. Given that during maximum exercise the latter vary within a very limited range [19], the higher O_2_ pulse values of the MAD deficient subjects must correspond to a greater stroke volume. In agreement with this view, it has recently been shown that CHF patients with CT + TT genotype of *AMPD1* C34T polymorphism have an elevated left ventricular ejection fraction compared with CC genotype [32]. Finally, while further studies are required to establish the respective parts of increased stroke volume and arterio-venous difference, our results support the view of a greater efficiency of the cardiovascular system to deliver O_2_ in MAD deficient subjects. The lower lactate and RER values in MAD subjects [6,16,33] suggest a greater reliance on oxidative energy production. Our results are in agreement with previous studies showing an improvement of performance in endurance events in athletes harbouring the *AMPD1* common mutation [34]. Since only a few part of *AMPD1* C34T carriers exhibit muscular complaints, the clinical relevance of this myopathy has been questioned [35]. As a reconciling alternative, one can hypothesized that the positive effect of *AMPD1* C34T on cardiovascular function counterbalances the detrimental effects on skeletal muscle. From an evolutionary perspective, the beneficial effect of *AMPD1* common mutation on cardiovascular function may therefore explain the high allele frequency in the general population.

Several limitations need to be considered. First, given the nature of the studied population, the generalizability of the present findings to CHF patients is a crucial issue. Additional studies in subjects with CHF, using both muscle biopsies and data from CPX performed without HR modulators, are required. Furthermore, the observed correlation between MAD activity and MCR is based on three categories of MAD activity using histoenzymology. One might argue that the quantitative measurement of MAD activity using biochemical assay may provide a better correlation with MCR. Our segmentation of abnormal MAD activity into two subgroups according to p-NBT staining is in agreement with the methodology of Tarnopolsky et al. [6] using an absent MAD activity group with no MAD staining and a decreased MAD activity group with low staining to study the pathophysiology of MADD. It has been shown that low MAD staining in cryosections is correlated to reduced in vitro MAD activity in biochemical assays [6,36,37]. The potential for bias was also reduced through the comparison of each patient to biopsy specimens from different patients analyzed at the same time. Further, the Spearman coefficient we used to calculate the level of correlation between MAD activity and MCR is robust, as a non-parametric method. Factors other than MAD activity may also influence the chronotropic response during exercise, such as medications and medical conditions. However, common causes of CI have been ruled out in the study design and recruitment, and the similar age in the different groups minimizes the impact of the age-related decrease in maximal heart rate. We also acknowledge that the plasma levels of adenosine were not measured in this study, although the MCR was used as a functional marker of adenosine effects on heart. We wish to emphasize that adenosine determination in blood samples is challenging, owing to a very short half-life in plasma due to rapid transformation by deamination to inosine by adenosine deaminase, or by phosphorylation to AMP by adenosine kinase [38]. Additionally, in this “adenosine concept”, it is not completely clear whether adenosine is produced by skeletal muscle itself or cardiac muscle in MAD deficiency. Since the earlier study by Sabina *et al*. showing an increased adenosine production by skeletal muscle in MAD deficient subjects [8], the increase in blood adenosine levels was subsequently attributed to skeletal muscle [2]. A breakthrough in these studies came from Kalski *et al*. [9], who showed that, using cardiac muscle biopsy in CHF patients, MAD activity is decreased in C34T *AMPD1* carriers. This suggests an in situ production of adenosine in heart in C34T *AMPD1* carriers with ensuing paracrine protective effects on coronary circulation and chronotropic response. Finally, it should also be pointed out that the desensitization of adenosine receptors is less prone to occur in MAD deficient subject [39], since adenosine production is increased only during exercise [8, 9].

## Conclusion

Our study shows that the metabolic-chronotropic response is decreased in reduced skeletal muscle MAD activity. This finding reveals a biological mechanism that links *AMPD1* gene to cardiac function.

## Supporting information

S1 File Dataset. (XLSX)

S1 Fig Fig 2 inset. O_2_ pulse is determined by stroke volume (SV), hemoglobin content (Hb) and arteriovenous oxygen saturation difference.
(DOC)

## Abbreviations

CPX: Cardiopulmonary Exercise Testing
MAD: Myoadenylate Deaminase
MCR: Metabolic-Chronotropic Relationship
