# Supplementary material for: Effects of AMPD1 common mutation on the metabolic-chronotropic relationship: Insights from patients with myoadenylate deaminase deficiency
Source: PLoS One. 2017 Nov 2;12(11):e0187266. doi: 10.1371/journal.pone.0187266 (PMC5667816; doi:10.1371/journal.pone.0187266)
Supplement: Supplementary file 1 [file pone.0187266.s002.doc]

$$O_2\,pulse = SV * 1.34 * Hb * (SaO_2 - S\bar{v}O_2)$$
